# Supplementary material for: Metabolic Pathway Pairwise-Based Signature as a Potential Non-Invasive Diagnostic Marker in Alzheimer’s Disease Patients
Source: Genes (Basel). 2023 Jun 17;14(6):1285. doi: 10.3390/genes14061285 (PMC10298314; doi:10.3390/genes14061285)
Supplement: Supplementary file 1 [file genes-14-01285-s001.zip › Supplenmentary Figures.pdf]

Metabolic Pathway Pairwise-based signature as a potential non-invasive diagnostic marker in AD patients

Yunwen Feng[1, †], Xingyu Chen[1, †], Xiaohua Douglas Zhang[2,*], Chen Huang[1, *]

[1] Dr. Neher's Biophysics Laboratory for Innovative Drug Discovery, State Key Laboratory of Quality Research in Chinese Medicine, Macau University of Science and Technology, Taipa, Macao SAR, 999078, China
[2] Department of Biostatitics, College of Public Health, University of Kentucky, Lexington, KY 40536, USA.

[*] Authors to whom correspondence should be addressed.
[†] These authors contributed equally to this work

Supplementary Figures

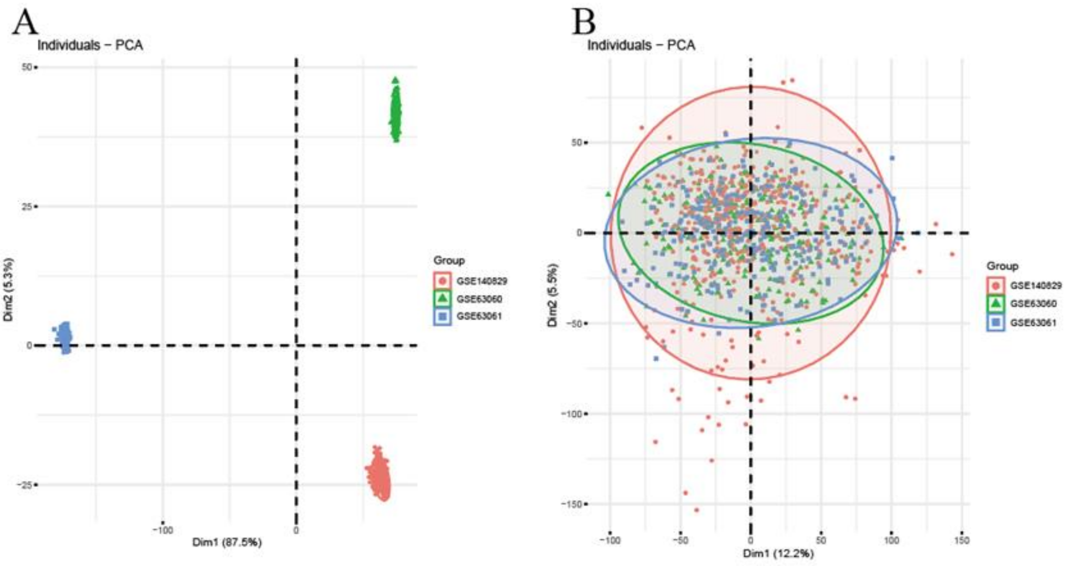

Figure S1. The PCA figures depict the datasets (GSE140829, GSE63060, and GSE63061) before and after removing the potential batch effect. (**A**) PCA scatter plot of three datasets before removing batch effect; (**B**) PCA scatter plot of three datasets after removing batch effect.

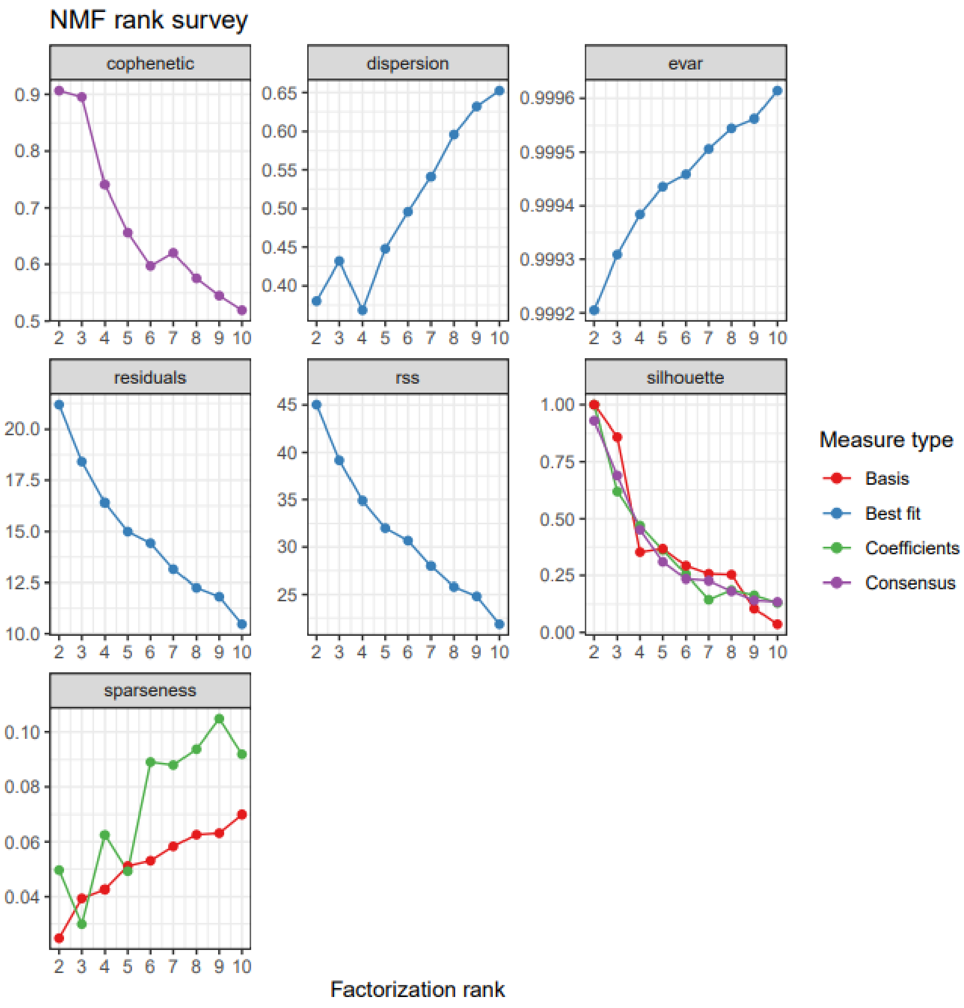

Figure S2. The cophenetic, dispersion and silhouette indicators determining the optimal clustering number of NMF method. The estimation is based on Brunet's algorithm. According to the Silhouette Coefficient, the clustering performance is best when there are 2 clusters. NMF, Non-negative Matrix Factorization.

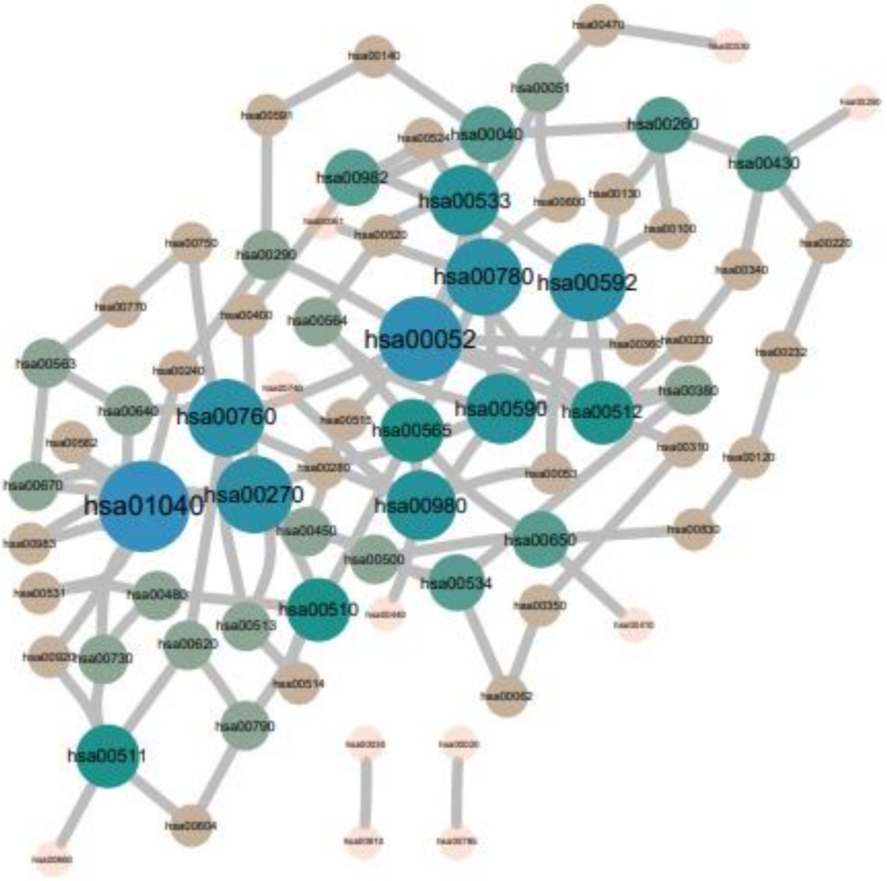

Figure S3. The metabolic network of differential MPP signatures between AD and non-AD groups revealing key metabolic pathways related to AD. We used 112 differential MPP signatures generated by chi-square test (adjusted p value < 0.01) between the AD and non-AD groups for metabolic network construction. Hub nodes in the metabolic network have deeper color and larger size. MMP, metabolic pathway pairwise.

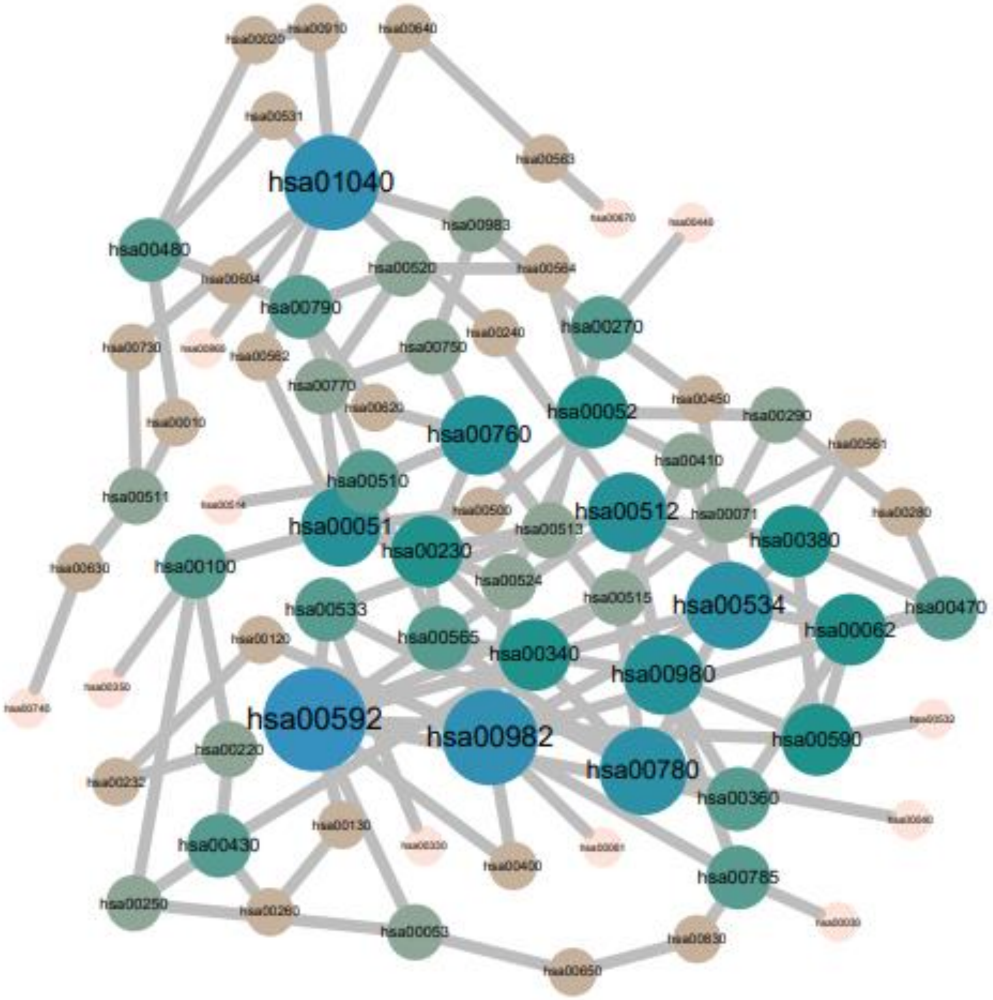

Figure S4. The metabolic network of differential MPP signatures between S1 and S2 groups revealing important metabolic pathways related to AD. We used 120 differential MPP signatures generated by chi-square test (adjusted p value < 0.01) between the S1 and S2 groups for metabolic network analysis. The deeper color and larger size reveal hub nodes in the metabolic network.
